# Collagenous Colitis with Escitalopram Use: A Case Report and Literature Review

**DOI:** 10.3390/healthcare12030330

**Published:** 2024-01-27

**Authors:** Emily Gray, Sara A. Wettergreen

**Affiliations:** Skaggs School of Pharmacy and Pharmaceutical Sciences, University of Colorado, Aurora, CO 80045, USA

**Keywords:** case report, collagenous colitis, escitalopram, microscopic colitis, SSRI

## Abstract

We present the case of a 42-year-old female whose escitalopram use potentially contributed to a diagnosis of collagenous colitis. The patient presented with significant watery, nonbloody diarrhea, abdominal cramping and pain, and weight loss. Established risk factors of microscopic colitis in this patient include a history of smoking and female gender. The patient underwent a colonoscopy, which confirmed histological changes consistent with collagenous colitis. Prescribed therapy included oral budesonide and omeprazole, continued for eight and twelve weeks, respectively. Escitalopram was continued, with a discussion regarding changing to an alternative therapy. Based on the patient’s history of escitalopram use, this case suggests a relationship between escitalopram and microscopic colitis. Though case reports of patients diagnosed with microscopic colitis after antidepressant use are published, this case appears to be the only report of collagenous colitis without macroscopic complications following escitalopram use. This case adds further support in that antidepressants may contribute to microscopic colitis. Despite an undefined frequency of association, healthcare providers who prescribe antidepressants should be cognizant of the theorized association and understand risk factors, screening, and treatment approaches.

## 1. Introduction

Microscopic colitis presents in two forms—lymphocytic or collagenous colitis. Both subtypes of microscopic colitis are a form of inflammatory bowel disease (IBD) [1]. Regardless of the subtype, patients with microscopic colitis will often present with watery diarrhea, abdominal pain and cramping, fecal urgency, fecal incontinence, and unintentional weight loss [1,2]. Differences in these subtypes are in histologic findings on biopsy [1,2]. While the exact mechanism of disease progression is still unclear, there are defined risk factors theorized to be linked to microscopic colitis [1,2]. These include concomitant diagnoses of autoimmune disorders, smoking, and age [1]. Females are more likely to be diagnosed than men [1]. Additionally, certain medication classes have been linked to microscopic colitis. Specifically, non-steroidal anti-inflammatory drugs (NSAIDs) and proton pump inhibitors (PPIs) have confirmed association, while antidepressants have a strong association with microscopic colitis [2].

This literature review and case report provides additional evidence to support the link between microscopic colitis and selective serotonin reuptake inhibitor (SSRI) use. Published case reports describe cases of lymphocytic and collagenous colitis associated with the use of SSRIs, serotonin and norepinephrine reuptake inhibitors (SNRIs), and tricyclic antidepressants (TCAs). However, there have been no published reports of collagenous colitis without macroscopic complications in patients who used escitalopram. In this report, we describe a patient whose depression had been managed with escitalopram for over five years before she began experiencing altered bowel habits, ultimately leading to a diagnosis of collagenous colitis.

## 2. Materials and Methods

**Figure 1:**
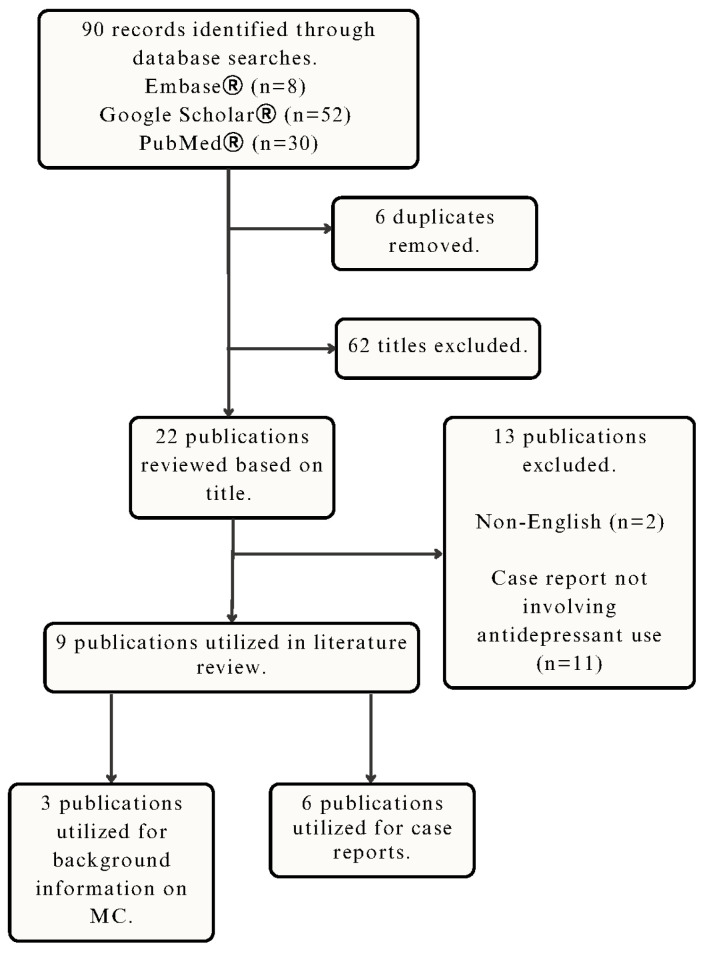
Literature search process and included studies.

This literature review aims to summarize all reported cases that suggest a relationship between antidepressant use and microscopic colitis. A search was conducted on Embase^®^, Google Scholar^®^, and PubMed^®^ using the following keywords and MeSH terms: microscopic colitis, collagenous colitis, SSRI, antidepressant, and escitalopram. Other terms were searched, specifically the names of other antidepressants. All non-English articles were excluded. With this search strategy, one result was found with escitalopram use and collagenous colitis; however, this patient also had macroscopic complications [3]. One case of lymphocytic colitis after escitalopram use was identified [3]. In total, six case reports (seven cases) were eligible for this literature review (Figure 1).

## 3. Results

### 3.1. Detailed Case Discussion

This case is a 42-year-old female who presented to her primary care office for a yearly follow-up and concerns regarding altered bowel habits. The patient reported symptoms of watery, non-bloody diarrhea (up to 30 episodes per day), unintentional weight loss, left upper quadrant (LUQ) cramping, and abdominal pain. Additionally, she reported amenorrhea for about 5 months. Her past medical history includes hypertension, prediabetes, polycystic liver disease, polycystic kidney disease, depression, cerebral aneurysm, and uterine fibroids. Relevant family history includes ulcerative colitis (mother) and Crohn’s disease (unknown family member). She has no known medication allergies. The patient is married, a former smoker (2.5 pack-year history), and has one alcoholic drink per week. She denies having tried any interventions to relieve her symptoms. Upon physical exam, tenderness of the LUQ and a midline mass extending from the subdiaphragmatic region to the umbilicus were noted. The patient’s depression has been well-controlled (PHQ-9 score of 1; GAD-7 score of 3) with escitalopram 20 mg by mouth once daily since 2018. She first noticed changes in bowel habits three months prior to the visit with her primary care provider (PCP), who referred the patient to gastroenterology. Within one month, she had a colonoscopy performed and met with gastroenterology to review the results. The colonoscopy confirmed a diagnosis of collagenous colitis. She also underwent an upper endoscopy to rule out celiac disease. Gastroenterology prescribed oral budesonide and omeprazole. It was also recommended that she connect with her PCP to discuss alternative antidepressants. 

Alternative diagnoses considered include infectious causes of diarrhea, H. pylori gastritis, celiac disease, Crohn’s disease, and ulcerative colitis. Diagnostic tests include a stool sample for infectious causes, a colonoscopy with random colon biopsies, an upper endoscopy with duodenal biopsies, H. pylori screening, celiac laboratory tests, and an abdominal MRI. The findings from the stool sample, H. pylori screening, duodenal biopsies, and abdominal MRI were negative and showed unremarkable findings. The celiac laboratory tests were positive for two genes associated with celiac disease (DAGA IgG and DQ2 serologies); however, the duodenal biopsy confirmed the patient is negative for celiac disease.

### 3.2. Treatment Outcomes

Prescribed treatments were omeprazole 20 mg by mouth twice daily and budesonide EC 9 mg by mouth once daily. The patient has not used any self-care interventions for symptom relief. Omeprazole was recommended to be used for 12 weeks. The duration of budesonide use was not defined. At the most recent encounter, she had completed omeprazole therapy and finished approximately eight weeks of budesonide. Her abdominal pain and cramping had resolved, but she was still having 5–10 episodes of diarrhea per day. 

At the time of this report, the patient has not followed-up with her gastroenterologist or PCP. During a visit with her nephrologist for unrelated issues, she reported having continued symptoms of microscopic colitis. It was also recommended that she discuss alternative antidepressants with her PCP, as escitalopram is theorized to have caused collagenous colitis. However, it does not appear that this has been discussed with a provider yet. Her continued use of escitalopram is potentially contributing to the persistence of her symptoms.

## 4. Discussion

### 4.1. Literature Review

We identified seven case reports of patients who were diagnosed with microscopic colitis while taking an antidepressant, as summarized in Table S1 [3,4,5,6,7,8]. Of these case reports, there were three cases of lymphocytic colitis, three cases of collagenous colitis, and one case of mixed lymphocytic and collagenous colitis. One author presented two cases of collagenous colitis where both patients also had macroscopic colitis complications (rectal ulcers) [3]. At the time of diagnosis, patients in the case reports were taking the following regimen(s): escitalopram, venlafaxine plus duloxetine, sertraline, venlafaxine, duloxetine, or nortriptyline (changed to duloxetine). Six of the seven case reports are female patients. Additional microscopic colitis risk factors present in the cases include a history of smoking, concurrent use of PPIs, and recent use of NSAIDs [3,4,6,8]. The duration of antidepressant use prior to a diagnosis of microscopic colitis varied. Menon and colleagues described a case of a 63-year-old male who took sertraline for two weeks prior to symptom development, while Iqbal and colleagues described the case of a 42-year-old female who had been taking escitalopram for over six months prior to symptom development. In the cases with reported treatment and outcomes, patients either discontinued the contributing antidepressant at the time of diagnosis or were recommended to switch to alternative antidepressants [3,4,6,8]. The most commonly reported treatment (aside from discontinuing or switching the contributing antidepressant) was oral budesonide. Four cases had successful outcomes with the use of oral budesonide [3,4,8]. Varelas and colleagues did not report on the treatment or outcomes. Interestingly, Varelas and colleagues reported on the case of mixed microscopic colitis (collagenous and lymphocytic). This patient presented without diarrhea, making it unique from the other cases [5]. In five cases, treatment with budesonide was successful in achieving a full remission of symptoms [4,7,8]. One case report describes unsuccessful use of budesonide, ultimately leading the patient to discontinue all psychiatric medications and use dicyclomine and sucralfate for symptomatic control [6]. Ultimately, this patient was able to start olanzapine and nortriptyline seven months after discontinuing other psychiatric medications and achieving symptomatic remission [6]. 

These case reports provide examples of the diverse onset of symptoms associated with microscopic colitis. The lack of distinct timing to symptom onset can be a barrier for healthcare providers when determining the etiology of a patient’s gastrointestinal symptoms. Additionally, there is not a consistent, well-documented management strategy for microscopic colitis. The first step is generally to discontinue the offending agent, which in the cases above is the antidepressant (SSRI, SNRI, or TCA). This is a challenge, as eliminating these medication classes leaves limited remaining options for the treatment of anxiety and/or depression, and available evidence from case reports does not establish if certain agents within the classes have a lower risk for microscopic colitis than the others.

For symptomatic relief, four cases discuss the use of over-the-counter remedies, including bismuth subsalicylate and loperamide [3,4,6]. The dose and duration of these remedies were not defined. In all four case reports, the patients reported unsuccessful symptom management with bismuth subsalicylate and loperamide. In 2015, the American Gastroenterological Association published guidelines for the management of microscopic colitis [9]. These guidelines primarily focus on prescribed medication therapy and non-medication interventions, such as oral budesonide. The 2021 European Guidelines on Microscopic Colitis describe that the consistent use of PPIs, NSAIDs, or SSRIs is associated with an increased risk of microscopic colitis; however, it is noted that this does not imply causality [10]. The 2021 guidelines recommend considering the withdrawal of any drug with a suspected relationship to the onset of diarrhea, along with medication therapy and non-medication interventions. 

One small clinical trial investigated the benefit of bismuth subsalicylate in microscopic colitis symptom management [11]. In this open-label trial, twelve participants with microscopic colitis were given bismuth subsalicylate 262 mg chewable tablets and were instructed to take eight tablets per day for eight weeks. Eleven of the twelve participants reported symptomatic improvement within eight weeks. Of note, there is no mention of risk factors or theoretical causes of microscopic colitis in the study participants. Given the small number of participants in the study, and that the study is over twenty years old, these results provide some, but minimal, evidence to support the use of bismuth subsalicylate in providing symptomatic relief for microscopic colitis. The American Gastroenterological Association guidelines from 2015 briefly discuss the use of bismuth subsalicylate, but no large clinical trials have demonstrated proven benefits in symptom management. However, based on the limited evidence available, bismuth subsalicylate could be used as a second-line alternative pharmacologic treatment if contraindications or financial burdens exist with corticosteroids [9]. It is noteworthy that the dose of bismuth subsalicylate mentioned in the guidelines is eight to nine tablets divided three times daily, which could present a significant pill burden. 

It has been well documented that neurotransmitters play a role in the gastrointestinal tract [12,13]. These neurotransmitters include norepinephrine, dopamine, serotonin, histamine, and gamma-aminobutyric acid (GABA) [14]. Serotonin, or 5-hydroxytryptamine (5-HT), is a neurotransmitter that plays a role in both the enteric nervous system (ENS) and central nervous system (CNS) [13]. In the gastrointestinal tract, serotonin plays a role in absorption, regulation of transport, and the release of fluids. Primarily, 5-HT is synthesized in the intestinal epithelium by enterochromaffin cells (EC). 

Research has been conducted to investigate the connection between serotonin reuptake transporter (SERT) mRNA in patients with active Crohn’s disease or ulcerative colitis [15]. They found that there appears to be reduced SERT present in the endothelium in patients who have active disease [14]. Given that microscopic colitis is a form of IBD, the reduced activity of SERT may also play a role in microscopic colitis. Because SERT is inhibited by SSRIs, this reduction could explain the mechanism of microscopic colitis when patients are exposed to SSRIs. 

SSRIs work by inhibiting the reuptake of serotonin, ultimately resulting in higher serotonin levels in the CNS. Escitalopram and other SSRIs stimulate all 5-HT receptors, including 5-HT3, which is primarily found in the gastrointestinal tract [16]. This stimulation results in increased gut motility, which can cause adverse effects, including nausea, vomiting, abdominal cramping, and diarrhea. Although frequently a transient effect, some patients may experience these gastrointestinal adverse effects until discontinuation of the SSRI [16]. The exact mechanistic link between SSRI exposure and microscopic colitis is unclear; however, the impact on 5-HT3 receptors ultimately causes irritation to the gastrointestinal tract, which can contribute to microscopic colitis [12]. Other possible causes of microscopic colitis include disruptions in the microbiota, autoimmune diseases, and improper absorption of bile acid. Serotonin may also impact the regulation of bile acid in the gastrointestinal tract [16]. More specifically, peripheral serotonin induces the contraction of the gallbladder, which releases bile acids into the gastrointestinal tract [16]. Although more research is needed, it can be proposed that the stimulation of 5-HT3 receptors and serotonin’s role in bile acid secretion contribute to the development of microscopic colitis secondary to SSRI exposure.

### 4.2. Contextualization

Both SSRIs and SNRIs are common first-line treatment options for adults and older adults with depression and/or anxiety [17]. It is important for healthcare providers prescribing SSRIs or SNRIs to be cognizant of the proposed risk of microscopic colitis, especially in patients with underlying risk factors of microscopic colitis. Patients who develop microscopic colitis may have debilitating symptoms that impact the activities of daily living and could lead to worsening psychiatric symptoms, as seen in one presented case report [6]. Additionally, if patients present with classic symptoms of microscopic colitis, prompt action should be taken, including consideration of discontinuing or switching the current antidepressant(s). 

Healthcare providers who prescribe SSRIs should be careful in distinguishing the anticipated gastrointestinal adverse effects and microscopic colitis symptoms. Again, the gastrointestinal adverse effects related to SSRI use are often mild and transient and include diarrhea and nausea [18]. The timing of symptom onset or worsening should also be taken into consideration, as dose escalations may temporarily worsen the adverse effects. Certain agents in the class, such as sertraline, have a higher risk for gastrointestinal adverse effects compared to other agents [18]. As discussed in the above literature review, the time of onset of microscopic colitis varies, making it difficult to determine when microscopic colitis should be considered in relation to SSRI initiation. However, persistent diarrhea, in addition to other microscopic colitis symptoms, should encourage healthcare providers to include microscopic colitis in their differential diagnoses.

### 4.3. Strengths and Limitations

The presented case describes the first report of collagenous colitis without macroscopic complications in a patient taking escitalopram, adding to the current literature supporting antidepressant use as a risk factor for microscopic colitis development. Given that the patient continues to have symptoms despite treatment suggests a relationship between escitalopram use and microscopic colitis symptom presentation. The literature review also explores the connection between serotonin and gastrointestinal symptoms, proposing a theoretical link between SSRI exposure and microscopic colitis development.

The presentation of collagenous colitis in the setting of escitalopram use seen in this case may not yet exist in the literature because of underreporting rather than the uniqueness of this case specifically. There are also potential confounding variables in this patient case, given her additional risk factors for microscopic colitis, including female sex and smoking history. Additionally, the literature review was limited, as there are very few case reports of microscopic colitis related to antidepressant use. Even more seldom are cases of collagenous colitis with SSRI use. The small sample size of published cases limits our ability to either confirm or deny the relationship between antidepressants and microscopic colitis. The published case reports and the presented case do not offer long-term follow-up on the patients’ psychiatric or gastrointestinal symptoms. Therefore, it is unclear whether the described therapeutic interventions offer a long-term resolution of microscopic colitis versus temporary symptomatic relief.

## 5. Conclusions

Microscopic colitis is a form of IBD that presents with significant symptoms, including watery, non-bloody diarrhea, abdominal pain and cramping, and fecal incontinence. Although the frequency is not defined, there appears to be an association with microscopic colitis onset and the use of antidepressants. In the presented case, we describe a patient with a history of escitalopram use who was diagnosed with collagenous colitis over five years after starting escitalopram. This appears to be the only case of collagenous colitis associated with escitalopram use without any macroscopic complications. In patients who present with classic microscopic colitis symptoms, healthcare providers should refer the patient for a colonoscopy to confirm diagnosis. Patients with a confirmed diagnosis of microscopic colitis should be switched to an alternative antidepressant and started on oral budesonide. This case report and literature review provides additional support that SSRI use is a risk factor for developing microscopic colitis. Further research is needed to define the risk of microscopic colitis with antidepressant use.

## Data Availability

No additional data is available for this case report and literature review.

## Supplementary Materials

The following supporting information can be downloaded at https://www.mdpi.com/article/10.3390/healthcare12030330/s1, Table S1: Literature summary of antidepressant-related colitis case reports.
