# Supplementary material for: Collagenous Colitis with Escitalopram Use: A Case Report and Literature Review
Source: Healthcare (Basel). 2024 Jan 27;12(3):330. doi: 10.3390/healthcare12030330 (PMC10855906; doi:10.3390/healthcare12030330)
Supplement: Supplementary file 1 [file healthcare-12-00330-s001.zip › healthcare-2729143-supplementary.pdf]

Table S1. Literature summary of antidepressant-related colitis case reports.

Reference, year of publication	Age, sex, reported risk factors	Antidepressant History	Patient-Reported Symptoms	Past Medical History	Laboratory & Clinical Findings	Diagnosis	Treatment	Outcomes
Iqbal et al., 2022 [4]	42, F<br><br>Former smoker	Escitalopram	Watery diarrhea (2-3 episodes per day); 2 episodes of fecal incontinence; nausea.<br><br>Onset appeared random with no obvious pattern.	High-grade small bowel obstruction; depression; anxiety	Colonic mucosa with notable inflammation; increased intraepithelial lymphocytic infiltrates.	Lymphocytic colitis	Pre-diagnosis: loperamide without relief.<br><br>Budesonide 9 mg PO once daily x 90 days.<br><br>Find alternative SSRI.	After 3 months, the patient showed significant symptomatic improvement and was tapered off budesonide therapy. Colonoscopy repeated and showed normal mucosa.
Varelas et al., 2022 [5]	45, F	Venlafaxine and duloxetine	Nausea; weight loss; NO diarrhea	Major depressive disorder	Colon mucosa biopsy showed mild increase in cell content; increased eosinophil presentation; intraepithelial lymphocytes; mild thickening of subepithelial collagen bands	Mixed colitis found in the esophagus, stomach, duodenum, and colon	No information on treatment reported.	No information on follow-up or outcomes reported.
Rahman et al., 2017 [3]	67, F	Escitalopram	Watery diarrhea	Not reported.	Solitary rectal ulcer; acute	Collagenous colitis with	Pre-diagnosis: loperamide with	Resolution of diarrhea and

	Concomitant PPI use				neutrophilic exudate; increased subepithelial collagen	macroscopic complications.	mild relief.<br><br>Budesonide (dose not reported).<br><br>Discontinuation of escitalopram and lansoprazole.	mucosal ulceration had resolved after two months.
Rahman et al., 2017 [3]	62, F	Venlafaxine	Non-bloody diarrhea (up to 15 episodes per day); epigastric pain; nausea	Major depressive disorder	Antral gastritis; collagenous thickening of basement membrane; rectal ulcer	Collagenous colitis with macroscopic complications.	Budesonide (dose not reported).<br><br>Venlafaxine was discontinued.	Symptomatic resolution.
Salter et al., 2017 [6]	50, F<br><br>Smoker<br><br>Recent NSAID use	Nortriptyline (tapered off while duloxetine started).<br><br>Duloxetine augmented with quetiapine and buspirone.	Watery diarrhea (up to 20 times per day); fecal incontinence; mild abdominal pain; nausea; bloating; > 9 kg weight loss	Depression with anxiety; chronic pain; chronic tobacco use; dental problems; two hospitalizations for psychiatric episodes.	Stool test: few lymphocytes. Random biopsies on colonoscopy confirmed lymphocytic colitis.	Lymphocytic colitis	Pre-diagnosis: loperamide and bismuth subsalicylate without relief.<br><br>Budesonide; tapered off duloxetine over 10 days.<br><br>Sucralfate and dicyclomine.<br><br>Discontinued all psychiatric medications.	Partial improvement with budesonide and after tapering off duloxetine. Still having 5-10 episodes of diarrhea per day.<br><br>Switched from budesonide to dicyclomine and sucralfate.<br><br>Still only mild improvement. Elected to stop all psychiatric and GI medications.

								Underwent ECT with modest mood improvement.<br><br>After 7 months, she started on olanzapine. A few weeks later, she added back nortriptyline. Achieved remission of depression and diarrhea.
Ranit et al., 2015 [7]	63, M	Venlafaxine (stopped after 1 week due to nausea)<br><br>Sertraline	Watery diarrhea; 15 kg weight loss over 4 weeks	Brief anxiety and depressive episode lasting for 3-4 months	Mild hypokalemia; lymphocytes in stool; fluid in large bowel on CT scan	Lymphocytic colitis	Sertraline tapered and discontinued over 48 hours.	Stopping sertraline fully resolved diarrhea within 2 weeks.
Sisman et al., 2012 [8]	66, F<br><br>Smoker	Duloxetine	Recurrent diarrhea; cramping; abdominal pain	Major depression	Tenderness on LLQ; mononuclear cell infiltration and collagen accumulation in bad style on pathology reports from biopsies; decreased hemoglobin; elevated ESR;	Collagenous colitis	Budesonide (dose not reported).<br><br>Cessation of duloxetine.	Diarrhea decreased by 50% after stopping duloxetine. Addition of budesonide completely resolved diarrhea.

					elevated CRP			

CRP = C-reactive protein; ECT = electroconvulsive therapy; ESR = erythrocyte sedimentation rate; LLQ = left lower quadrant; LUQ = left upper quadrant; NSAID = non-steroidal anti-inflammatory drug; PPI = proton pump inhibitor.
